# Ganglioside Antibody‐Mediated Autoimmune Unilateral Sciatic Neuritis

**DOI:** 10.1002/ccr3.71145

**Published:** 2025-10-03

**Authors:** Andreas Posa, Andreas Schlüter, Malte E. Kornhuber

**Affiliations:** ^1^ University Clinic and Outpatient Clinic for Radiology Martin Luther University Halle‐Wittenberg Halle (Saale) Germany; ^2^ Radiological Clinic HELIOS Hospital Sangerhausen Sangerhausen Germany; ^3^ University Clinic and Outpatient Clinic for Neurology Martin Luther University Halle‐Wittenberg Halle (Saale) Germany; ^4^ Neurological Clinic HELIOS Hospital Sangerhausen Sangerhausen Germany

**Keywords:** autoimmune neuropathy, diffusion‐weighted MRI, ganglioside antibodies, immunoglobulin therapy, sciatic neuritis

## Abstract

Autoimmune‐mediated neuropathies may cause lumbar pain. Therefore, an extended diffusion‐weighted MRI scan and autoimmune ganglioside antibody diagnostics should be performed to detect rare autoimmune‐mediated inflammatory and demyelinating peripheral neuropathies and to treat them with immunomodulatory therapies, especially in cases of long‐standing and therapy‐resistant symptoms.

## 1 Case Presentation

A female patient (35a) presented with 6 months of lumbar pain (pulling, electrical, load‐dependent, radiating from the lumbar spine into the left leg). The initial treatment was carried out by an orthopedic surgeon who suspected a lumbar disc problem and lumbar muscle tension. Regular x‐rays and magnetic resonance imaging (MRI) (T1 and T2 weighted) of the lumbar spine showed no significant abnormalities. Physiotherapy and analgesic medication (initially non‐steroidal anti‐inflammatory drugs: ibuprofen: 400 mg 3× daily; then switched to paracetamol: 500 mg 4× daily) had no significant effect. Because of this pain, the patient had difficulty walking and climbing stairs.

Six months after the onset of symptoms, the patient was referred to our neurological clinic because of persistent discomfort and additional paralysis. The left foot elevation showed a 4/5 paresis according to MRC (*Medical Research Council*) and the left great toe elevation was 2/5. No other neurological complaints were noted, either subjectively or objectively. In summary, the peroneal part of the sciatic nerve was clinically affected. There were no further indications of other differential diagnoses (e.g., L5 radiculopathy or fibular head syndrome) based on clinical, electrophysiological or radiological findings. The pain described above was rated at this time on the Numerical Rating Scale between 2 (at rest) and 8 (on exertion). Electrocardiogram, electromyography, electroneurography and cerebrospinal fluid examination were unremarkable. Serology for *Borrelia burgdorferi* in cerebrospinal fluid and blood serum was negative.

**FIGURE 1:**
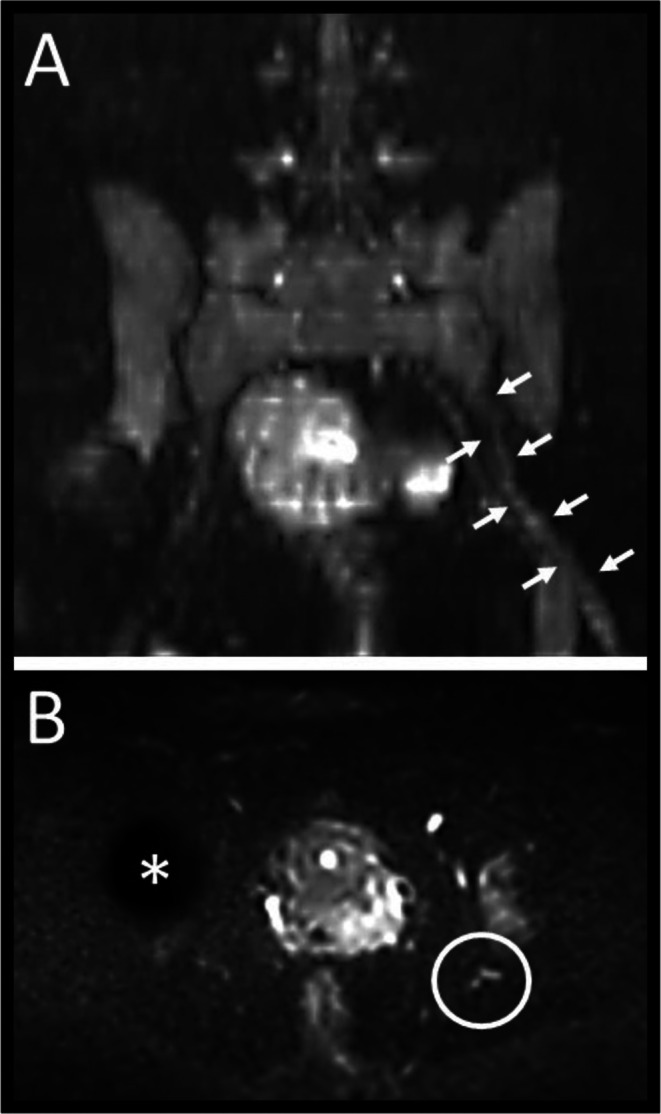
Diffusion‐weighted MRI scans of the lumbar spine showed a thickened and signal‐altered left sciatic nerve in the diffusion weighting (A: Frontal plane, arrows; B: Transversal plane, circle). Star: Artifact due to total hip arthroplasty.

Diffusion‐weighted MRI of the lumbar spine showed a thickened and long‐term hyperintense diffusion restriction of the left sciatic nerve (Figure 1) originating from the greater sciatic notch. Antibody diagnostics revealed clearly pathological levels of the ganglioside antibodies GQ1b (259%; cut‐off 30%) and GT1a (427%; cut‐off 30%) (normal: GM1, GD1a, GD1b) [1].

We diagnosed ganglioside antibody‐mediated autoimmune unilateral sciatic neuritis as a subvariant of inflammatory demyelinating peripheral neuropathy and started immunomodulatory therapy with immunoglobulins (induction phase: 0.4 g/kg body weight intravenously per day for 5 days) [2]. This resulted in a complete resolution of the symptoms. The cause of this autoimmune reaction remained unclear. There was no evidence of previous viral infection or other immunological triggers.

In our rare case of autoimmune‐mediated isolated nerve damage, diffusion‐weighted MRI was helpful in localizing the ischial nerve lesion. Diffusion‐weighted MRI is an appropriate functional imaging technique for visualizing peripheral nerve structures [3]. In cases of nerve injury or inflammation, the amount of water outside the nerve cells increases, and the collagen support structures are destroyed. Under these conditions, diffusion‐weighted MRI can detect thickening and a localized increase in signal intensity in the affected nerve.

This case is peculiar in that the isolated nerve lesion is presumably due to an autoimmune attack directed against ganglioside epitopes without other signs of chronic inflammatory demyelinating polyneuropathy or its rare subvariant multifocal acquired demyelinating sensory and motor neuropathy or Guillain–Barré syndrome. It may therefore be that the spectrum of autoimmune‐mediated anti‐ganglioside‐mediated neuropathies is broader than previously thought. This case also illustrates how misdiagnosed patients can be left mistreated for weeks or even months and suffer from their symptoms.

## Author Contributions

**Andreas Posa:** conceptualization, data curation, investigation, methodology, visualization, writing – original draft. **Andreas Schlüter:** investigation, methodology, writing – review and editing. **Malte E. Kornhuber:** formal analysis, investigation, resources, supervision, writing – review and editing.

## Consent

During her hospitalization, the adult patient gave her written informed consent on the hospital's internal data protection forms to the use of her patient‐related data in anonymized form for scientific publication.

## Conflicts of Interest

The authors declare no conflicts of interest.

## Data Availability

The clinical data related to this case report are available on request from the corresponding author. The data are not publicly available due to privacy and ethical restrictions.
